# Leptin Receptor Gln223Arg Polymorphism of Human Spermatozoa Associated with Male Infertility in a Chinese Population

**DOI:** 10.1155/2023/4009061

**Published:** 2023-08-23

**Authors:** Yi Mo, Fangfang Liang, Arshad Mehmood, Suleman Shah, Ying Xie, Zhong Lin, Yan Sun, Hesheng Jiang, Yafen Guo, Xiangli Niu, Dinggan Mo

**Affiliations:** ^1^The Reproductive Hospital of Guangxi Zhuang Autonomous Region, Nanning 530029, China; ^2^Guangxi University of Chinese Medicine, Nanning 530200, China; ^3^Department of Neurology, The Second Hospital of Hebei Medical University, Shijiazhuang 050000, Hebei, China; ^4^Department of Cell Biology and Genetics, School of Basic Medical Sciences, Health Science Center, Shenzhen University, Shenzhen, China; ^5^Department of Genetics, Hebei Medical University, Hebei Key Lab of Laboratory Animal, Shijiazhuang 050017, Hebei, China; ^6^College of Animal Science and Technology, Guangxi University, Nanning 530005, China

## Abstract

**Background:**

Leptin (LEP) is believed to play a crucial role in male reproduction, while the molecular mechanisms through which LEP affects the male reproductive system are unclear. LEP acts by binding to a leptin receptor (LEPR) which mediates its physiological action, but there are only limited studies on the function of LEPR in human sperm.

**Purpose:**

This study aimed to determine the Gln223Arg polymorphisms of the LEPR gene in human spermatozoa and evaluate their possible relationship with semen variables.

**Methods:**

The study was performed on Chinese men: 115 healthy subjects and 108 patients with primary and 98 with secondary infertility. Semen samples were obtained from all patients, and semen variables were analyzed. The genotypic and allelic frequencies of Gln223Arg polymorphism in spermatozoa were determined by PCR and restriction fragment length polymorphism (RFLP) analyses. Statistical analyses were performed using the chi-square test, the Kruskal–Wallis test, and the Mann–Whitney test.

**Results:**

There were no significant differences in genotypic or allelic frequency distributions of Gln223Arg polymorphism among men with primary infertility, secondary infertility, and controls. Similarly, semen volume and sperm concentration did not differ with the different genotypes in all groups of men. The percentages of motile sperm for AA + AG genotypes in men with primary infertility (31.98%) were significantly lower than those in secondary infertility, and control men with GG genotypes were 34.41% and 59.36%, respectively. At the same time, the percentages of normal morphology sperm for AA + AG genotypes in men with primary infertility (2.93%) were significantly lower than those in secondary infertility and control men with GG genotypes 3.71% and 6.54%, respectively.

**Conclusion:**

This study reveals a possible association between the Gln223Arg polymorphism of the LEPR gene in spermatozoa affecting spermatozoal membrane integrity and having a direct role in sperm motility.

## 1. Introduction

Infertility affects approximately 10% to 15% of couples, and male factors account for 40% of infertility cases [1]. Despite an increasing effort to identify the causes of male infertility, infertility research has received much less attention than most common, complex diseases [2]. LEP is believed to play a crucial role in male reproduction, as plasma concentrations were negatively correlated with a variety of reproductive indices. The overexpression of LEP in hyperleptinemic mice decreased the weight and volume of the testicles, the diameter of the seminiferous tubules, and the numbers of the spermatocytes, spermatozoa, Leydig cells, and offspring. After hyperleptinemia withdrawal, the testicular structure and function were partially recovered. These results indicated hyperleptinemia adversely affected testicular development, function, and fecundity [3]. LEP deficiency caused defects in spermatogenesis in Akita mice, resulting in infertility. After LEP treatment, the Akita mice revealed larger overall testes and seminal vesicles than untreated Akita sibling mice, their sperm motility was significantly increased, and spermatogenesis was restored. The results showed that LEP treatment could prevent degeneration of the testes [4]. LEP has been suggested to pharmacologically reduce the effects of obesity on male fertility, although data on its potential use in clinical practice are still limited [5]. The rs10244329 polymorphism in the LEP gene showed a statistically significant difference in fertility; it appeared that variability in the LEP gene might be associated with male infertility [6]. Studies have suggested that LEP indirectly affects male reproductive function via the central neuroendocrine system and directly via the peripheral tissue membrane receptors [7]. The molecular mechanisms through which LEP affects the male reproductive system are elusive [8]. In target tissues, LEP acts by binding to a LEPR which mediates its physiological action. Soluble LEPR has been found in human-seminal plasma, and plasmalemmal LEPR exists in the interstitium of testicular tissue, testis, and sperm [9–11]. A 145 kDa isoform of LEPR was localized by immunofluorescence microscopy to the tail region of ejaculated spermatozoa [9, 12]. The tail of spermatozoa contributes mainly to sperm motility. In this regard, studies suggested a possible role for LEP signaling in sperm motility. In cases of idiopathic asthenozoospermia, sperm motility was negatively associated with concentrations of LEP in seminal plasma but not in the serum. This also suggested that LEP exhibited certain local effects in the testis [13]. LEPR is encoded by the LEPR gene, which has at least six different isoforms. These LEPR isoforms result in different biological activities of LEP. The genetic variation and expression of the LEPR might affect male fertility; thus, the possible role requires further research [6]. Knock-out LEPR mice have small testes, azoospermia, and multinucleated spermatids, while patients with LEPR gene mutations are infertile [14–16]. Since 2009, as a novel approach, single nucleotide polymorphism (SNP) arrays have provided valuable data on rare genetic variations in men with impaired spermatogenesis [17]. Through peripheral blood samples for DNA extraction and genotypic analysis, the association between polymorphisms of LEP and LEPR genes and male infertility has been examined [6, 18]. The mechanistic basis for the regulatory role of LEP signaling in male infertility, especially between LEPR polymorphisms of spermatozoa and male infertility, awaits further analysis. The Gln223Arg (rs1137101) polymorphism of LEPR, the most studied in human subjects, lies within the first domain in two putative LEP-binding regions and may affect all forms of the receptor.

The present study was conducted to examine the Gln223Arg polymorphism of LEPR in human spermatozoa and evaluate its association with semen variables.

## 2. Materials and Methods

### 2.1. Study Population and Sample Collection

A total of 321 males participated in this study and were referred to Guangxi Research Center for Family Planning (Nanning, China). Samples of patients were collected from June 2017 to December 2018. The study population was selected based on the following criteria: (1) body mass index (BMI), follicle-stimulating hormone (FSH), luteinizing hormone (LH), testosterone, and prolactin levels were normal; (2) volunteers with infertility causes, such as thyroid disorder, inflammation, and infectious disease, varicocele, and abnormal karyotype were excluded; (3) those with female factor infertility were excluded. All participants were assigned to one of three groups: primary infertility (*n* = 108), secondary infertility (*n* = 98), and controls (*n* = 115). Primary infertility patients (aged 26–40 years) referred to a man for whom the culprit factor cannot identify and who had no child after at least a year of unprotected intercourse. Secondary infertility patients (aged 25–40 years) are defined as having the inability to have a child for at least 12 months after having had children. The healthy donors (aged 24–39 years) in the control group gave birth to children by natural conception with their female partners, and the criteria for selecting healthy fertile individuals included normal genital examinations and normozoospermia in addition to the following criteria: sperm concentration ≥15 × 10^6^/ml, total motility ≥40%, progressive motility ≥32%, and normal forms ≥4%. Each participant signed informed consent to participate in the study, which was approved by the Ethical Committee of the Guangxi Research Center for Family Planning. Semen samples were collected in the laboratory of the Guangxi Research Center for Family Planning and from all participants by masturbation after 3–5 days of sexual abstinence.

### 2.2. Semen Analysis and Spermatozoa Preparation

Semen variables were examined after liquefaction according to the World Health Organization laboratory manual [19]. Semen volume was measured with a 10 mL serological pipet. The Sperm Class Analyzer system (Microptic S.L., Barcelona, Spain) was employed to determine sperm concentration (×10^6^/mL), sperm motility (%), and straight-line velocity (VSL) (*μ*m/s). Semen smears stained with Berg's stain (carbol fuchsin and methylene blue) were used to detect sperm abnormalities. After semen analysis, the remaining raw semen was filtered through glass wool to remove gelatinous material, and then, sperm was obtained by centrifugation twice at 1000 g for 10 minutes (min) and resuspension in Earle's balanced salt solution (EBSS). Finally, spermatozoa were purified by the swim-up method (SUM).

### 2.3. DNA Extraction and Genotyping of LEPR Gln223Arg Polymorphisms

Sperm genomic DNA was extracted using the standard phenol-chloroform method, and its quality was determined by the ratio of A260 : A280. The extracted DNA was stored at 4°C until used for amplification of a 416 bp fragment bracketing the LEPR Gln223Arg polymorphism and genotyped by RFLP analyses. For PCR amplification, the following primers, based on Khosropour et al. [18], were used (Table 1).

**Figure 1:**
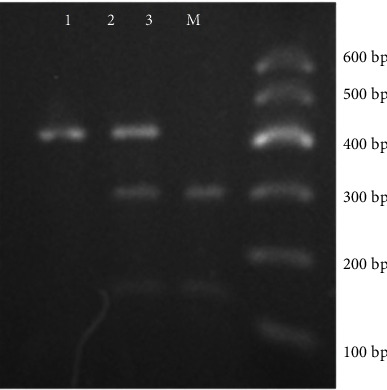
A representative 3% agarose gel shows RFLP products of LEPR after digestion with MspI: lane 1 is the AA genotype (416 bp); lane 2 is the AG genotype (416, 291, and 125 bp); lane 3 is the GG genotype (291 and 125 bp); lane M is DNA ladder.

DNA was amplified on a Bio-Rad S1000 PCR machine (Hercules, CA) according to the following protocol: initial denaturation at 95°C for 3 min, followed by 35 cycles of 95°C for 30 seconds (sec), 53°C for 30 sec, and elongation at 72°C for 30 sec, and a final extension at 72°C for 5 min. After the size of PCR products (416 bp) was confirmed by electrophoresis on 2% agarose gels, PCR products were subjected to restriction enzyme (Msp 1) digestion at 37°C overnight, according to the manufacturer's protocol (Thermo Scientific, Waltham, MA). Restriction fragments containing ethidium bromide were electrophoresed on 3% agarose gels and detected visually under ultraviolet light. Thus, to evaluate the accuracy of genotyping, a random double-typing approach was used on 20% of the restriction fragments derived from PCR products. The verification process resulted in a 100% match, indicating high accuracy in the genotyping analysis. The restriction fragments revealed three genotypes (Figure 1). The AA genotype (absence of restriction site) indicated a single 416 bp fragment, and the GG genotype exhibited two fragments of 291 and 125 bp, while the AG genotypes exhibited all three fragments, including 416, 291, and 125 bp.

### 2.4. Statistical Analysis

All data were analyzed using the Statistical Analysis System (SAS) software (version 9.2). The gene counting method was used to calculate the allelic frequencies, and the distribution of genotypes for Hardy–Weinberg equilibrium was tested in all groups. The Chi-square test (*χ*^2^) was used to analyze the significance of the association between allelic and genotypic frequencies and infertility status. After applying one-sample Kolmogorov–Smirnov tests, the data of semen variables showed nonnormal distribution; therefore, an equivalent nonparametric test was used. The significance of the association between semen parameters and genotypes was analyzed using the Kruskal–Wallis test, and the descriptive characteristics of the group variables were expressed as median with an interquartile range. Thus, when statistical significance was demonstrated (*P*  <  0.05), comparisons between groups were made with the Mann–Whitney test. This study revealed more than 80% power to detect the genotypic association of LEPR polymorphism with a significance level of 0.05 using online tools, such as “https://www.power.com” and “https://sample.size.com.”

## 3. Results

### 3.1. Leptin Receptor Gln223Arg Polymorphism Analysis

The genotypic distribution and allelic frequencies of the Gln223Arg polymorphism are presented (Table 2). Genotypic distribution in all groups was in accordance with the Hardy–Weinberg equilibrium. Allelic and genotypic frequency distributions of G1n223Arg polymorphisms among men with primary infertility, secondary infertility, and controls were compared using Chi-square tests (Table 3). The results indicate that no significant differences in genotypic or allelic frequency distributions were detected for Gln223Arg polymorphism in the three groups.

### 3.2. Semen Variables in Genotypes of LEPR Gln223Arg

Semen variables in men with primary infertility, secondary infertility, and controls distributed by genotypes of LEPR Gln223Arg polymorphisms are presented (Table 4). There were no significant differences in semen volume and sperm numbers. Sperm motility of AA + AG genotypes in primary infertility was significantly lower than that of the GG genotype in the three groups. The sperm motility of AA + AG genotypes was significantly lower than that of the GG genotype in controls. Sperm normal morphology of AA + AG genotypes in primary infertility was significantly lower than that of the GG genotype in all three groups. Sperm normal morphology of AA + AG genotypes in secondary infertility and controls was significantly lower than that of the GG genotype in controls.

## 4. Discussion

The LEPR gene includes 20 exons and exists as several common variants, including 2 nonconservative changes at codon 223 in exon 6, the glutamine to arginine substitution (CAG to CGG) (Q223R), and at codon 656 in exon 14, the lysine to asparagine substitution (AAG to AAC) (K656N). There is a conservative change at codon 109 in exon 4, the lysine to arginine substitution (AAG to AGG) (K109R), and 2 silent changes in exon 1 at codon 1019 (CGT to CAT, Pro1019Pro) and at codon 343 (GTG to GCG, Ser343Ser) [20]. Among these, the A to G substitution at codon 223 (Gln223Arg polymorphism) is within the extracellular domain of LEPR and affects all forms of the receptor. This substitution could affect LEPR function and change its signaling capacity [21]. The present study examined the relationship between allelic and genotypic frequencies of the Gln223Arg polymorphism with male infertility in a Chinese population and analyzed semen variables to determine whether Gln223Arg polymorphism of LEPR was directly associated with male infertility.

In the present study, the LEPR 223Arg allelic frequencies of primary infertility, secondary infertility, and controls were 87.04%, 84.69%, and 83.48%, respectively. Although the LEPR 223Arg allelic frequencies were much higher than figures reported for Caucasians (44%), Pima Indians (32%), and Brazilians of European descent (40%), these estimates were comparable to others (85%) measured in Asians [22]. These tests and the regenotyping of 20% of the samples indicate the reliability of the data. The frequencies of GG, AG, and AA genotypes in Chinese men in these three groups differed from those of other populations, but the lack of difference in allelic and genotypic frequencies related to fertility status was consistent with findings in Iranian, Slovenian, and Macedonian men [6, 18]. Meanwhile, the Gln223Arg polymorphism and genotypic or allelic frequency distributions did not differ among individuals with primary infertility, secondary infertility, and controls.

For the semen variables, semen volume and sperm concentration in the different genotypes did not vary significantly with fertility status. In contrast, sperm motility and sperm normal morphology of AA + AG genotypes in primary infertility were significantly lower than those of GG genotypes; male primary infertility is more likely in carriers of the Gln (A) allele. The percentage of progressively motile sperm in Iranian men carrying the AA and AG genotypes was lower than that of those with the GG genotype of LEPR [18], whereas another study involving Slovenians and Macedonians [6] found no differences between genotypes with semen variables. These varied results undoubtedly reflect the studied populations having different ethnicities and genetic backgrounds.

As codon 223 in exon 6 of the LEPR gene lies within the first of two putative LEP-binding regions, an A to G transition mutation (Gln223Arg) could affect the affinity of LEP-binding affinity and impair the signaling capacity of the LEPR [23]. Serum LEP-binding affinity was affected by LEPR Gln223Arg polymorphism; it is lower in individuals carrying the Gln (A) allele than in those who were homozygous for the Arg (G) allele [24]. Thus, Western blot and fluorescence microscopy indicated that LEPR in spermatozoa was significantly related to the integrity of the spermatozoal membranes. The quantity of LEPR in spermatozoa with deteriorated membranes was significantly lower than in those with intact membranes [9]. In addition, some studies showed that the (A) allele of LEPR Gln223Arg gene polymorphism might be associated with type 2 diabetes mellitus (T2DM) [25]. As the leading cause of T2DM, obesity may induce testicular oxidative stress (OS), while fatty acid oxidation produces large amounts of ROS due to adipose tissue accumulation. Thus, the amounts of ROS may mediate oxidative damage in the sperm membrane and affect sperm DNA integrity [26]. In the present study, the BMI of the selected study population was normal, and oxidative damage is less likely to damage the sperm membrane. The A to G transition mutation (Gln223Arg) of LEPR in this Chinese population might reduce LEP-binding affinity in individuals carrying the Gln (A) allele and thus lower the percentage of motility sperm. Meanwhile, spermatozoal LEPR in individuals with AA + AG genotypes was significantly related to the intactness of the spermatozoal membranes, likely reducing the percentage of sperm with normal morphology.

Genes encode proteins, and proteins are the embodiment of specific genes. Alternative splicing of the human LEPR mRNA can generate several isoforms of the protein: the full-length form (OB-Rb), soluble form (OB-Re), and short forms (OB-Ra, OB-Rc, OB-Rd, and OB-Rf) [27–29]. The activation of these receptors can convey different biological activities of LEP. The various isoforms are not uniform across all tissues, and their presence and position, as well as function in human sperm, need further studies [30]. It is planned to determine which forms of the LEPR protein exist in spermatozoa of the stated Chinese individuals with the Gln223Arg polymorphism and examine their relationship with sperm motility and morphology.

## 5. Limitations

This study has several potential limitations which should be acknowledged. First, a small sample size of our study is not enough to get the optimal statistical power to detect a correlation with the size of the weak effect; thus, the results of our research should be further verified in a larger cohort. Second, social and psychological data of patients were not obtained, such as success in the history of marriage or childbirth, lack of sexual knowledge, and disharmony of married life; these issues could lead to a heavy psychological burden that causes infertility. Finally, information on patients' lifestyles, such as work environment, career, and dietary habits, was not available; these factors would also impact male fertility status.

## 6. Future Perspectives

Based on this study, we are expected to expand the sample size and verify the corresponding results. Then, we consider whether LEPR Gln223Arg polymorphism in spermatozoa could be used as the biomarker included in the screening program for male infertility, especially in obese men. However, the mechanism of how the LEPR Gln223Arg mutation in spermatozoa may influence the integrity of the membranes from spermatozoa should be explored in future studies.

## 7. Conclusion

Result reveals lower percentages of motility sperm and those with normal morphology in AA + AG genotypes than in GG genotypes in Chinese men with primary infertility, regardless of fertility status. Our findings suggest that the LEPR Gln223Arg polymorphism in spermatozoa may be associated with the integrity of the membranes from spermatozoa and play a direct role in sperm motility to cope with the limits of LEPR, also with less unintended results.

## Figures and Tables

**Table 1:** Primers are used for PCR amplification.

Name	Sequence (5′-3′)
Forward primer	CCCTTTAAGCTGGGTGTCCCAAATAG
Reverse primer	GCTAGCAAATATTTTTGTAAGCAATT

**Table 2:** Genotype distribution and allelic frequencies of Gln223Arg polymorphism in the LEPR gene.

Groups	*n*	Allelic frequency		Genotypic frequency			*P* value (*χ*^2^, df)
		A	G	AA	AG	GG	
Primary infertility	108	0.1296	0.8704	0.009	0.241	0.75	0.8970 (*χ*^2^ = 0.0168, df = 1)
Secondary infertility	98	0.1531	0.8469	0	0.306	0.694	0.7197 (*χ*^2^ = 0.1288, df = 1)
Controls	115	0.1652	0.8348	0.017	0.296	0.687	0.8707 (*χ*^2^ = 0.0265, df = 1)

Only proportions are given for the alleles and genotypes. ^*∗*^*P* value <0.05 for differences after the Hardy–Weinberg equilibrium test.

**Table 3:** Allelic and genotypic frequency distributions of G1n223Arg polymorphisms among subjects with primary infertility, secondary infertility, and controls.

	Primary infertility, *n* (%)	Secondary infertility, *n* (%)	Controls, *n* (%)	*χ* ^2^ (*P*)
Genotypes				
AA	1 (0.9)	0 (0.0)	2 (1.7)	
AG	26 (24.1)	30 (30.6)	34 (29.6)	
GG	81 (75)	68 (69.4)	79 (68.7)	1.2601 (0.5326)
AA + AG	27 (25)	30 (30.6)	36 (31.3)	
Alleles				
A	12.96	15.31	16.52	0.5160 (0.7726)
G	87.04	84.69	83.48	

The number of cases and proportions (in parentheses) is given for the genotypes. Only proportions are given for the alleles. The *P* value represents the Chi-square analysis of genotypes (AA + AG and GG) and alleles (A and G) by groups. *P*  <  0.05 for differences among primary infertility, secondary infertility, and controls.

**Table 4:** Semen variables of primary infertility, secondary infertility, and controls partitioned by genotypes of LEPR Gln223Arg polymorphisms.

Semen variables	Primary infertility	Secondary infertility		Controls			*P* value
	AA + AG (*n* = 27)	GG (*n* = 81)	AA + AG (*n* = 30)	GG (*n* = 68)	AA + AG (*n* = 36)	GG (*n* = 79)	
Semen volume (ml)	3.99 (2.6–5.1)	3.27 (1.8–4.55)	3.52 (2.4–4.3)	3.25 (2.1–4.25)	3.10 (2.2–4.05)	3.35 (2.2–4.2)	0.1977
Sperm counts (million/ml)	40.91 (12–42.7)	29.54 (2–41.05)	58.07 (26.3–80.9)	53.94 (17.25–65.65)	62.31 (39.3–80.2)	69.38 (33.5–90.6)	0.5753
Sperm motility (%)	31.98^*∗*^ (23.2–39.1)	34.54 (19.8–52.1)	32.96 (27.2–38.7)	34.41 (23.65–45.35)	56.12^*∗*^ (43.65 - 66.15)	59.36 (50–68)	*P* < 0.0001
Sperm normal morphology (%)	2.93^*∗*^ (1–4)	3.12 (1–5)	4.97^*∗*^ (3–7)	3.71 (2–6)	5.94^*∗*^ (4–7)	6.54 (5–8)	*P* < 0.0001

^*∗*^ *P* value <0.05. ^*∗*^Significant difference between AA + AG and GG among primary infertility, secondary infertility, and controls. Results are presented as median (interquartile range).

## Data Availability

The datasets used during the current study are available from the corresponding author upon reasonable request.

## Additional Points

(i) Missense mutations in the LEPR gene are associated with changes in semen quality. (ii) A novel missense mutation in the LEPR gene (Gln223Arg) with male infertility in a Chinese population is determined by PCR-RFLP technology. (iii) There are no significant differences in genotypic or allelic frequency distributions of Gln223Arg polymorphism among different groups. (iv) The percentages of motile sperm and those with normal morphology are significantly lower for AA+AG genotypes in men with primary infertility than those in men with GG genotypes. (v) Gln223Arg polymorphism of the LEPR gene in spermatozoa affects spermatozoal membrane integrity and has a direct role in sperm motility.

## References

[1] Devroey P., Fauser B. C., Diedrich K. (2009). Approaches to improve the diagnosis and management of infertility. *Human Reproduction Update*.

[2] Nuti F., Krausz C. (2008). Gene polymorphisms/mutations relevant to abnormal spermatogenesis. *Reproductive BioMedicine Online*.

[3] Yuan M., Huang G., Li J. (2014). Hyperleptinemia directly affects testicular maturation at different sexual stages in mice, and suppressor of cytokine signaling 3 is involved in this process. *Reproductive Biology and Endocrinology*.

[4] Schoeller E. L., Chi M., Drury A., Bertschinger A., Esakky P., Moley K. H. (2014). Leptin monotherapy rescues spermatogenesis in male Akita type 1 diabetic mice. *Endocrinology*.

[5] Alves M. G., Jesus T. T., Sousa M., Goldberg E., Silva B. M., Oliveira P. F. (2016). Male fertility and obesity: are ghrelin, leptin and glucagon-like peptide-1 pharmacologically relevant?. *Current Pharmaceutical Design*.

[6] Hodžić A., Ristanović M., Zorn B. (2017). Genetic variation in leptin and leptin receptor genes as a risk factor for idiopathic male infertility. *Andrology*.

[7] Sáinz N., Barrenetxe J., Moreno-Aliaga M. J., Martínez J. A. (2015). Leptin resistance and diet-induced obesity: central and peripheral actions of leptin. *Metabolism*.

[8] Abbasihormozi S., Shahverdi A., Kouhkan A., Cheraghi J., Akhlaghi A. A., Kheimeh A. (2013). Relationship of leptin administration with production of reactive oxygen species, sperm DNA fragmentation, sperm parameters and hormone profile in the adult rat. *Archives of Gynecology and Obstetrics*.

[9] Jope T., Lammert A., Kratzsch J., Paasch U., Glander H. J. (2003). Leptin and leptin receptor in human seminal plasma and in human spermatozoa. *International Journal of Andrology*.

[10] Ishikawa T., Fujioka H., Ishimura T., Takenaka A., Fujisawa M. (2007). Expression of leptin and leptin receptor in the testis of fertile and infertile patients. *Andrologia*.

[11] Zhang J., Jin P. P., Gong M., Yi Q. T., Zhu R. J. (2018). Role of leptin and the leptin receptor in the pathogenesis of varicocele-induced testicular dysfunction. *Molecular Medicine Reports*.

[12] Li H. W., Chiu P. C., Cheung M. P., Yeung W. S., O W. S. (2009). Effect of leptin on motility, capacitation and acrosome reaction of human spermatozoa. *International Journal of Andrology*.

[13] Guo J., Zhao Y., Huang W. (2014). Sperm motility inversely correlates with seminal leptin levels in idiopathic asthenozoospermia. *International Journal of Clinical and Experimental Medicine*.

[14] Mounzih K., Lu R., Chehab F. F. (Mar 1997). Leptin treatment rescues the sterility of genetically obese ob/ob males. *Endocrinology*.

[15] Clément K., Vaisse C., Lahlou N. (1998). A mutation in the human leptin receptor gene causes obesity and pituitary dysfunction. *Nature*.

[16] Bhat G. K., Sea T. L., Olatinwo M. O. (2006). Influence of a leptin deficiency on testicular morphology, germ cell apoptosis, and expression levels of apoptosis-related genes in the mouse. *Journal of Andrology*.

[17] Krausz C., Escamilla A. R., Chianese C. (2015). Genetics of male infertility: from research to clinic. *Reproduction*.

[18] Khosropour S., Hamidi M., Fattahi A. (2017). Leptin and leptin-receptor polymorphisms in fertile and infertile men. *Systems Biology in Reproductive Medicine*.

[19] Lu J. C., Huang Y. F., Lü N. Q. (2010). [WHO laboratory manual for the examination and processing of human semen: its applicability to andrology laboratories in China]. *Zhonghua Nan ke Xue*.

[20] Méndez-Sánchez N., Bermejo-Martínez L., Chávez-Tapia N. C. (2006). Obesity-related leptin receptor polymorphisms and gallstones disease. *Annals of Hepatology*.

[21] Cleveland R. J., Gammon M. D., Long C. M. (2010). Common genetic variations in the LEP and LEPR genes, obesity and breast cancer incidence and survival. *Breast Cancer Research and Treatment*.

[22] Paracchini V., Pedotti P., Taioli E. (2005). Genetics of leptin and obesity: a HuGE review. *American Journal of Epidemiology*.

[23] Okobia M. N., Bunker C. H., Garte S. J. (2008). Leptin receptor Gln223Arg polymorphism and breast cancer risk in Nigerian women: a case control study. *BMC Cancer*.

[24] Quinton N. D., Lee A. J., Ross R. J., Eastell R., Blakemore A. I. (2001). A single nucleotide polymorphism (SNP) in the leptin receptor is associated with BMI, fat mass and leptin levels in postmenopausal Caucasian women. *Human Genetics*.

[25] Adiga U., Banawalikar N., Mayur S., Bansal R., Ameera N., Rao S. (Apr 1 2021). Association of insulin resistance and leptin receptor gene polymorphism in type 2 diabetes mellitus. *Journal of the Chinese Medical Association*.

[26] Arafa M., Elbardisi H., Majzoub A., Agarwal A. (2020). *Genetics of Male Infertility: A Case-Based Guide for Clinicians*.

[27] Takaya K., Ogawa Y., Isse N. (1996). Molecular cloning of rat leptin receptor isoform complementary DNAs--identification of a missense mutation in Zucker fatty (fa/fa) rats. *Biochemical and Biophysical Research Communications*.

[28] Löllmann B., Grüninger S., Stricker-Krongrad A., Chiesi M. (1997). Detection and quantification of the leptin receptor splice variants Ob-Ra, b, and, e in different mouse tissues. *Biochemical and Biophysical Research Communications*.

[29] Tartaglia L. A. (1997). The leptin receptor. *Journal of Biological Chemistry*.

[30] Catteau A., Caillon H., Barrière P., Denis M. G., Masson D., Fréour T. (2016). Leptin and its potential interest in assisted reproduction cycles. *Human Reproduction Update*.

